# Autocrine and Paracrine Mechanisms Promoting Chemoresistance in Cholangiocarcinoma

**DOI:** 10.3390/ijms18010149

**Published:** 2017-01-13

**Authors:** Massimiliano Cadamuro, Simone Brivio, Carlo Spirli, Ruth E. Joplin, Mario Strazzabosco, Luca Fabris

**Affiliations:** 1 School of Medicine and Surgery, University of Milan-Bicocca, 20900 MB Monza, Italy; massimiliano.cadamuro@unimib.it (M.C.); s.brivio3@campus.unimib.it (S.B.); mario.strazzabosco@yale.edu (M.S.); 2 International Center for Digestive Health (ICDH), University of Milan-Bicocca, 20900 MB Monza, Italy; 3 Digestive Disease Section, Yale University School of Medicine, New Haven, CT 06520, USA; carlo.spirli@yale.edu; 4 The School of Medicine, Royal Derby Hospital, University of Nottingham, Derby DE22 3NE, UK; ruthjop@hotmail.co.uk; 5 Department of Molecular Medicine, University of Padova School of Medicine, 35131 PD Padova, Italy

**Keywords:** liver cancer, tumor reactive stroma, cancer stem cells, morphogens, apoptosis

## Abstract

Resistance to conventional chemotherapeutic agents, a typical feature of cholangiocarcinoma, prevents the efficacy of the therapeutic arsenal usually used to combat malignancy in humans. Mechanisms of chemoresistance by neoplastic cholangiocytes include evasion of drug-induced apoptosis mediated by autocrine and paracrine cues released in the tumor microenvironment. Here, recent evidence regarding molecular mechanisms of chemoresistance is reviewed, as well as associations between well-developed chemoresistance and activation of the cancer stem cell compartment. It is concluded that improved understanding of the complex interplay between apoptosis signaling and the promotion of cell survival represent potentially productive areas for active investigation, with the ultimate aim of encouraging future studies to unveil new, effective strategies able to overcome current limitations on treatment.

## 1. Introduction

Cholangiocarcinoma (CCA) is one of the most aggressive and lethal malignancies in humans. CCA comprises a heterogeneous group of liver cancers, originating from epithelial cells lining the bile ducts (cholangiocytes), including both the extrahepatic and intrahepatic segments. The extrahepatic form is more common, accounting for 80%–90% of CCAs, and depending on the location within the extrahepatic biliary system, this type is divided further into perihilar (Klatskin tumor) and distal [1,2]. Although deemed as a ‘rare’ tumor, the incidence of intrahepatic CCA (iCCA) specifically has steadily increased worldwide over the last few decades, with an increased incidence in males [3]. The highest rates are observed in Eastern Asia, in regions endemic for hepatobiliary fluke infestations (Thailand: up to 85 per 100,000). Progressive rises have also been reported in Western countries; CCA has reached an incidence of 1.67 per 100,000 in the USA, while in Europe, it ranges from 0.45 (Switzerland) to 3.36 (Italy) per 100,000 [3,4]. Based on these epidemiological data, CCA has currently become the second most common primary hepatic malignancy, after hepatocellular carcinoma (HCC), thus generating renewed interest of the scientific community towards this disease. Until recently, CCA has been little studied, largely because of the lack of experimental models, limited availability of tissue samples and a lower priority for research funding compared with other more common malignant diseases [5]. This has led to persistent gaps in knowledge, particularly in the field of therapy and treatment strategies. Radical treatment options for CCA remain limited to liver resection, but can only be offered to a minority of patients (20%–40% with iCCA) because of the propensity of CCA for early dissemination. Liver transplantation is available only for carefully-selected cases in just a few, highly-specialized liver centers [6,7]. Both surgical procedures are further complicated by high rates of recurrence [6,7] and the likely advanced stage of malignant disease at diagnosis. Prognosis for patients with CCA is therefore typically poor, with median survival of less than 24 months, and just 5% of the patients survive beyond five years from diagnosis. For patients ineligible for surgery, palliation that includes radiotherapy or stenting to relieve biliary obstruction may provide some benefit [6]. Pharmacological interventions have proved disappointing due to the high resistance of CCA to drug-induced cytotoxicity, so that until a few years ago, chemotherapy was generally excluded from the treatment algorithm for these patients [1,8]. Intrinsic resistance to drug cytotoxicity is a key feature of normal cholangiocytes since they are equipped with a rich repertoire of defense enzymatic activities that protect them from toxic compounds present in bile and/or hepatic blood [9]. Following malignant transformation, an additional protective role is played by the extensive desmoplastic microenvironment wherein the neoplastic ducts are embedded, termed tumor reactive stroma (TRS). Recent data support the concept that the TRS is a key determinant of decreased sensitivity of CCA to drug-induced cytotoxicity, hampering responses to chemotherapy and resulting in a poor clinical outcome. Resistance to chemotherapy thus represents a major issue in CCA treatment. Indeed, in contrast to HCC, where early detection of malignant change is a major goal of the cirrhosis follow-up process, CCA is often diagnosed at advanced stages, because of the lack of symptoms and the frequent absence of a liver disease background that demands ongoing routine clinical surveillance. For the newly-diagnosed CCA patient ineligible for surgery, palliation is often the only option apart from those allocated to specific clinical trials. Although chemotherapy has been classically recognized to be unsuccessful, gemcitabine has sometimes been prescribed in “off-label” regimens, based on its approved use in pancreatic cancer, together with fluoropyrimidines and cisplatin. A recent study of more than 400 patients with advanced CCA suggested that combined cisplatin and gemcitabine improved patient overall survival by around four months compared with patients undergoing gemcitabine alone, thus opening potential new pharmacological perspectives for patients ruled out from surgery [10].

Here, we present an in-depth analysis of autocrine and paracrine pro-survival signals originating in the tumor microenvironment. We attempt to apply reviewed data (based on a systematic literature search) to suggest how the avoidance of normal apoptotic mechanisms in cancer cells may cause chemoresistance in CCA.

## 2. Mechanisms of Chemoresistance

The lack of sensitivity of tumor cells to chemotherapeutic drugs is still a major cause of disease progression and mortality in cancer patients, especially in those with recurrent or advanced malignancy. Chemoresistance is generally categorized into two different groups: primary, developing at the start of any given treatment, and secondary (or acquired), occurring at a certain point of the treatment schedule, after a number of chemotherapeutic cycles [11]. CCA is paradigmatic of the malignant tumors with primary chemoresistance [12]. Mechanisms underpinning chemoresistance are multiple and affect drug metabolism (including uptake, export and intracellular biotransformation), the expression of specific molecular targets mediating drug effects on cancer cells, the ability of repair mechanisms to circumvent drug-induced DNA damage and the balance between pro- and anti-apoptotic factors [12].

Given the strong involvement of biliary epithelium in modifying bile composition through fluidification and alkalinization of the primary bile secreted by hepatocytes, cholangiocytes are constitutively endowed with multiple membrane carriers that mediate both the uptake and extrusion of many different molecules [13]. These transport activities enable cholangiocytes to regulate the intracellular levels of several xenobiotics of both an anionic and cationic nature, including anti-cancer drugs. For example, the organic anion-transporting polypeptides (OATP) and organic cationic transporters (OCT) are extensively expressed by cholangiocytes, where they mediate the uptake of methotrexate, taxanes and imatinib (OATP) [14,15], as well as of platinum and tyrosine kinase inhibitors (OCT) [16]. Additional cholangiocyte carriers are the nucleoside transporters, equilibrative and concentrative, regulating active transport of gemcitabine and 5-fluorouracil (5-FU) [17,18]. Recent data indicate that in CCA cells, these transporters can be variably down-modulated, thus decreasing the intracellular accumulation of these drugs. At their apical aspect, cholangiocytes possess a wide range of efflux pumps. These belong to the ATP-binding cassette (ABC) family of multidrug resistance (MDR) proteins, which regulate the extrusion of many substrates, including several chemotherapeutic agents, such as paclitaxel, against a concentration gradient [19]. Transporters expressed by cholangiocytes are finely and timely regulated at a transcriptional level by a class of specific transcription factors, the nuclear receptors, which provide an adaptive response to meet the variable physiological needs according to the bile and plasma levels of transported solutes. An example of this regulatory mechanism is the control of MDR expression by the farnesoid X receptor (FXR), the main nuclear receptor activated by bile acids. As bile acid concentration rises in the liver during cholestasis, FXR is bound by bile acids, and its transactivation leads to the upregulation of MDR. Since cholestasis commonly develops in CCA, this mechanism leads to MDR overexpression on tumoral bile ducts, thus enabling drug extrusion out of cancer cells [20]. A similar chemoresistant phenotype derived from ABC transporter overexpression is particularly relevant in breast cancer, where the first ABC transporter associated with chemoresistance was discovered (ABCB1/P-glycoprotein). Inhibition of ABCB1/P-glycoprotein has been proposed as a strategy to reverse the MDR phenotype, although its high toxicity profile has limited results [21].

To exert cytotoxic effects, several anticancer drugs necessitate metabolic activation, before being eventually inactivated by conversion into water-soluble metabolites amenable to excretion into bile or urine. Therefore, intracellular enzymatic functions promoting chemoresistance include either decreased activation of pro-drug compounds or enhanced inactivation of active metabolites. These activities are operated by biotransformation enzymes of Phases I and II, which are constitutively expressed in liver cells, but are present also in many cancer cell types. For instance, CCA cells express enzymes crucially regulating gemcitabine and 5-FU metabolism, acting as phosphorylase or monophosphate synthetase [22]. Drug metabolism is also dependent on the intracellular levels of glutathione (GSH), as occurs for cisplatin, which is conjugated with GSH by glutathione-*S*-transferase (GST) to be inactivated. Of note, GST is constitutionally expressed in cholangiocytes and further stimulated in CCA cells [23]. Increased expression of GST has been also reported in ovarian cancer, where it directly correlates with resistance to cisplatin.

A prerequisite of effective response to any given chemotherapeutic agent is the expression by cancer cells of the specific molecular target, which sustains its anticancer effect. If target expression is decreased, the drug cytotoxic effect is diminished. For instance, a reduced expression of topoisomerase-I (topo-I), involved in DNA replication, has been reported in colorectal and ovarian cancer cells resistant to irinotecan, acting through covalent binding to topo-I [24,25]. In CCA cells, expression of estrogen receptor (ER)-α and ER-β, whose activation modulates the balance between proliferation (mediated by ER-α) and apoptosis (mediated by ER-β) [26], can affect response to both tamoxifen, an ER antagonist [27], and KB9520, a selective ER-β agonist [28].

The mechanism of action of many anticancer drugs, such as cisplatin and 5-FU, relates to DNA damaging effects, resulting in the generation of bulky DNA adducts, which are responsible for cell cycle arrest and cell death. However, cells possess different strategies to repair DNA damage. Among them, the nucleotide excision repair (NER) pathway is one of the most extensively studied. The NER pathway leads to the activation of several endonucleases cleaving DNA adducts, such as the excision repair cross-complementing 1 (ERCC1), whose overexpression has been observed in several platinum-resistant cancer cells, including colorectal [25], ovarian [29], gastric [30] and CCA as well [31]. Notably, ERCC1 down-modulation in ovarian cancer cells by antisense oligonucleotides enhanced cisplatin cytotoxicity both in vitro and in in vivo xenograft models [32]. In addition to NER, there are other DNA damage repair mechanisms driven by RAD51 and DNA mismatch repair (MMR) proteins. RAD51 is a recombinase associated with low sensitivity to neoadjuvant chemotherapy in breast cancer [33], which has been found to be upregulated in CCA [34]. The MMR family is essential to maintain genomic stability, by scanning newly-synthesized DNA to detect single-base mismatches and nucleotide insertion-deletion errors [35]. *MMR* gene defects have been associated with cisplatin resistance, given the ability of MMR proteins to detect specific DNA adducts [36]. Mutations in *MMR* genes (*MSH2*, *MSH3*, *MSH6*, *hMLH1*, *PMS2*) have also been found in CCA, where they are associated with a stronger chemoresistant profile and worst outcome [12].

### Mechanisms of Chemoresistance Depending upon Evasion from Apoptosis

Altered sensitivity to drug-induced apoptosis plays a pivotal role in the chemoresistance of many malignant tumors and derives from an imbalance between anti-apoptotic (downregulated or inactivated) and pro-apoptotic (upregulated or aberrantly activated) signals. These signals are particularly susceptible to modulation by soluble factors (cytokines, chemokines, growth factors) that originate in the tumor microenvironment either from the tumor cell itself or from the surrounding stromal inflammatory cells. Both extrinsic (mediated by death receptors belonging to the tumor necrosis factor (TNF) receptor superfamily, such as Fas, and DR4/DR5) or intrinsic (mediated by mitochondrial proteins) pathways of apoptosis can be affected.

Among mitochondrial proteins, overexpression of anti-apoptotic members of the Bcl-2 family is a phenotypic trait frequently underlying sensitivity loss to anticancer drugs. Aberrant expression of the prototypical member Bcl-2 was originally described as a result of chromosomal translocation in follicular lymphoma, but subsequently, it was also frequently observed in solid tumors [37] and in CCA cell lines [38]. Notably, Bcl-2 overexpression is a defining feature of reactive cholangiocytes, an early histological marker for many non-neoplastic primary diseases of the biliary epithelium [39], thus indicating a constitutive ability of cholangiocytes to escape from apoptosis following liver injury. Among the proteins of the Bcl-2 family, myeloid cell leukemia (Mcl)-1 is an important pro-survival mediator and the major anti-apoptotic protein expressed in cholangiocytes [40], playing a key role in the MDR phenotype, which is amenable to selective inhibition by different compounds, such as maritoclax [41]. In non-small cell lung cancer, Mcl-1 downregulation reversed cisplatin resistance [42]. Interestingly, Mcl-1 expression is stimulated by bile salts, in particular by glycochenodeoxycholate, which hinders its proteasomal degradation by the ubiquitin E3 ligase through MEK-dependent phosphorylation [43]. This mechanism is likely relevant in cholestasis and, thus, in CCA. On the other hand, inactivation of Bax, a pro-apoptotic protein member of the Bcl-2 superfamily, has been observed in colorectal cancer, due to somatic loss-of-function mutations [44]. Whether expression levels of Bcl-2 and Bax actually correlate with response to chemotherapy is as yet uncertain, as shown in breast [45,46] and colorectal cancer [47]. However, upregulation of Bcl-2 in conjunction with downregulation of Bax is a signature of iCCA cells with resistance to cisplatin and 5-FU and depends on the activation of extracellular signal-regulated kinase (ERK)1/2 and AKT pathways [48]. Functional defects in the pro-apoptotic transcription factor p53, which upregulates both intrinsic (Bax) and extrinsic (Fas) mediators of apoptosis, were originally associated with decreased sensitivity to 5-FU, in vitro [49], but no current evidence supports p53 status as a determinant of response to anticancer drugs. Nevertheless, *TP53* mutations have been associated with poor outcome in CCA patients [50]. Defective activation of caspases is another mechanism frequently decreasing chemosensitivity in cancer cells. In this context, inhibitors of apoptosis proteins (IAPs) are a family of intracellular effectors, including X-linked inhibitor of apoptosis protein (XIAP) and survivin, whose pro-survival activity relies on their ability to bind active caspases (-3, -7 and -9). XIAP behaves as a potent chemoresistant factor in ovarian cancer [51], while survivin overexpression hampers drug-induced apoptosis in a variety of cultured cancer cells [52], and its expression levels correlate negatively with response to chemotherapy in ovarian [53] and esophageal cancer [54]. XIAP and survivin are both upregulated in CCA cells, where they induce chemoresistance [55,56].

Inhibition of extrinsic apoptosis may also contribute to chemoresistance in several cancer conditions. In this mechanism, death receptors are directly stimulated by ligand binding, e.g., Fas (CD95) by Fas ligand and DR4/DR5 by TNF-related apoptosis-inducing ligand (TRAIL). Their stimulation results in recruitment of adaptor proteins, such as Fas-associated death domain (FADD), and subsequent activation of initiator caspases (caspase-8, also known as FADD-like interleukin-1β-converting enzyme (FLICE), and caspase-10), which eventually activate pro-apoptotic effector pathways. Human cholangiocytes constitutively express both Fas [57,58] and DR5 [59]. A defective function of Fas and DR4/DR5 represents an additional mechanism of tumor chemoresistance. In fact, down-modulation of Fas expression has been reported in either hematologic (leukemia) or solid (neuroblastoma) malignancies with strong chemoresistance, whereas loss of TRAIL receptor expression has been extensively described in many common epithelial cancers, including breast, lung and HCC [60]. Of note, perturbations of TRAIL receptor delivery to the cell membrane from endoplasmic reticulum have been also observed in colon cancer cells [61]. In addition, some soluble factors may inhibit ligand binding to Fas, as nucleolin in B-cell lymphoma [62]. Alternatively, decoy receptors can be aberrantly expressed by cancer cells and allow them to escape from TRAIL-induced apoptosis, as TRAIL-R3 does in gastric carcinoma [63]. Upregulation of cellular FLICE-like inhibitory protein (c-FLIP) in cancer cells, preventing procaspase-8 processing, is a further inhibitory mechanism of death receptor-mediated apoptosis, which is relevant for chemoresistance in colorectal cancer [64] and HCC cells [65]. Similar to nucleolin, calmodulin exerts inhibitory effects on Fas-induced apoptosis by interacting with Fas, a possible mechanism of chemoresistance in CCA [66]. Notably, several of these anti-apoptotic proteins, including Bcl-2, Bcl-X_L_, IAPs and c-FLIP, are upregulated by the transcriptional activation of nuclear factor kappa B (NFκB) [67], thus highlighting a link between inflammation and chemoresistance. Indeed, NFκB is a key regulator of inflammation in many disease conditions, including cholangiopathies, and its inhibition enhances chemotherapy-induced apoptosis in epithelial cancers with intense stromal reaction, as shown in head-neck squamous carcinoma [68] and in pancreatic carcinoma [69].

Altogether, these mechanisms of chemoresistance are profoundly modulated by cues released in the tumor microenvironment, leading to a highly cross-talking, “multiethnic cellular society”. The fine arrangement of the TRS, including the multiple cell elements engaged in the complex interplay with the malignant epithelial counterpart, will be now reviewed.

## 3. Tumor Reactive Stroma

Excessive deposition of connective tissue that develops in conjunction with growth of tumor epithelial cells results in an integrated and well-tuned system, fed and sustained by complex mutual interactions. In CCA, this histopathologic feature, originally called desmoplasia or, more recently, TRS, is particularly prominent and profoundly affects how neoplastic ducts grow [70,71]. Unlike normal stroma, where a few quiescent fibroblasts lay within a physiological extracellular matrix (ECM), TRS contains a wide population of mesenchymal, inflammatory and immune cells embedded in a structurally-modified ECM. The TRS cell population includes perpetually-activated fibroblasts (termed cancer-associated fibroblasts (CAF)) [72], tumor-associated macrophages (TAM) [73], T cells, neutrophils and endothelial cells, within an ECM scaffold mainly enriched in fibrin and collagen type I fibers [72]. Paracrine or endocrine signals consist of growth factors, cytokines, chemokines and proteases released in a dysregulated fashion without temporal control. These factors actively contribute to the generation of the tumor microenvironment, encouraging an extensive cross-talk among the different cell types. Importantly, degradation of the basement membrane where transformed duct cells reside is a prerequisite permissive of the extensive web of communications in this region [71]. In contrast with the common mechanisms governing tissue repair and remodeling after tissue injury, the TRS may be regarded as an excessive “wound healing” [74], aimed at supporting epithelial cell survival, proliferation, motility and invasion [71]. Besides being a defining characteristic of CCA, an abundant TRS is also found in other highly invasive epithelial cancer types with pronounced multidrug resistance properties, such as pancreas [75], breast [76], ovary [77] and prostate [78]. Whilst a number of studies, both in vitro and in vivo, have highlighted the importance of the cooperation between neoplastic and stromal cells in promoting tumor progression [79,80], the ability of TRS cells to induce cancer cell resistance to chemotherapy has emerged only recently [81,82].

### 3.1. Cancer-Associated Fibroblasts

CAF, the major cellular component of the TRS, play a critical part in all steps of carcinogenesis, from neoplastic transformation to tumor dissemination [72]. Their cell source has been the subject of intense debate, since in the liver they may derive from hepatic stellate cells, portal fibroblasts or circulating bone marrow-derived mesenchymal cells; hypothetical origins via epithelial-to-mesenchymal transition (EMT) have however now been ruled out [83,84,85]. CAF are recruited close to the epithelial compartment and are persistently activated by the wide range of soluble factors produced by the tumor cells themselves, as well as by the multiple inflammatory cell types populating the TRS. Among these factors, platelet-derived growth factor (PDGF)-D, transforming growth factor (TGF)-β, reactive oxygen species (ROS) via secretion of nitric oxide (NO) and fibroblast growth factor (FGF)-2 are those more extensively characterized [86]. Once in close proximity to transformed cells in tumoral ducts, CAF favor tumor growth as a result of overexpression of hepatocyte growth factor (HGF), heparin-binding epidermal growth factor (HB-EGF) [87], TGFβ and stromal cell-derived factor (SDF)-1 (also known as CXCL12) [71]. Indeed, HGF, EGF and SDF-1 stimulate the migratory capabilities of CCA cells by activating ERK-1/2 and Akt [88]. On the other hand, CAF can further recruit monocytes, macrophages, endothelial cells and inflammatory cells to the TRS, given their ability to secrete numerous growth factors, such as vascular endothelial growth factor (VEGF) and FGF, coupled with a variety of cyto/chemokines, including monocyte chemoattractant protein (MCP)-1 (also known as CCL2), SDF-1 and interleukin (IL)-1. Moreover, CAF are able to promote cancer invasiveness by eliciting structural changes in the ECM supporting the TRS. These structural alterations include upregulation of neuropilin-1, a co-receptor and signaling amplifier of various VEGF family members, the matrix metalloproteinases (MMP)-1, -2 and -9, facilitating matrix degradation associated with the progression of many cancer types, and periostin and tenascin C [89,90]. Periostin and tenascin C are two highly reactive ECM components, which induce the expression of integrins α5β1 and α6β4, transmembrane heterodimeric receptors mediating cell-cell and cell-ECM interactions [89]. Their upregulation in neoplastic cholangiocytes leads to the activation of the phosphoinositide 3-kinase (PI3K)/AKT signaling pathway, which eventually stimulates escape from apoptosis and cell invasion. In addition, CAF may also affect the chemoresistance of CCA cells, an effect dependent on the activity of periostin, prostaglandin E2 (PGE2), sphingosine-1-phosphate and PDGF-B. In this context, the ECM may further support these CAF-mediated pro-tumorigenic effects, acting as a reservoir of soluble factors, which are then supplied continuously [71].

### 3.2. Tumor-Associated Macrophages

Tumor-associated macrophages (TAM) are M2-polarized macrophages (mainly deriving from circulating monocytes) that infiltrate the tumor microenvironment and promote tumor progression [73]. While M2 macrophages are involved in tissue remodeling, as well as in angio- and lymphangio-genesis, M1 macrophages seem to mainly exert tumor suppression functions via a high production of T-cell stimulatory cytokines [73]. TAM infiltration is associated with a poorer prognosis in many cancer types, including CCA [91]. However, recent data indicate that a subset of TAM with a mixed M1/M2 phenotype localized at the tumor periphery is actually involved in CCA progression, and molded by the cancer stem cell (CSC) niche [92]. TAM derive from circulating monocytes, which are recruited into the substance of the tumor by a wide range of chemokines, especially MCP-1/CCL2, CXCL1, CXCL10 and SDF-1/CXCL12, released either by tumor cells or other stromal cells [73,93]. Once drawn into the tumor, monocytes differentiate into M2 macrophages upon stimulation of many soluble mediators, such as PGE2, IL-2, IL-10 and TGF-β1 secreted by CAF and other inflammatory cells [71]. Although TAM density correlates with a poor outcome after surgical resection, their precise pro-oncogenic effects remain uncertain. They possibly depend on the stimulation of tumor cell motility and invasion, by various factors differentially involved in angiogenesis and ECM remodeling. For instance, colony stimulating factor (CSF-1), granulocyte-macrophage colony stimulating factor (GM-CSF), VEGF, FGF-1/2, PDGF, insulin-like growth factor (IGF)-1, TGF-β, IL-1, IL-6, IL-8, leukemia inhibitory factor (LIF), prostaglandins, interferon (IFN)-γ, TNF-α and MMPs have been reported [71,94]. Recent findings suggest that in CCA, TAM-derived CCL18 and CXCL9 may direct the self-renewal and drug-resistance properties of the CSC compartment [92].

### 3.3. Endothelial Cells

Another structural component of the TRS that in CCA is particularly prominent is the lymphatic vasculature. In CCA, lymphatic vessels are likely the main root of the metastatic spread. In this neoplasia, an extended and highly branched lymphatic bed nearby CAF and neoplastic ducts is a common histological picture. Both CAF and TAM are sources of specific lymphangiopoietic growth factors, such as VEGF-A, -C, -D and angiopoietin-1 and -2, as reported in breast cancer [95,96,97]. In contrast with the marked expansion of the lymphatic vasculature, blood vessels are significantly reduced in CCA compared with HCC, thus contributing to the hypoxic milieu in CCA [85]. Of note, hypoxia is a potent driver of tumor invasiveness via activation of the hypoxia inducible factor (HIF)-1α, the expression levels of which directly correlate with tumor size and inversely correlate with disease-free and overall survival in iCCA [98]. Moreover, HIF-1α stimulated PDGF-D expression by CCA cells, promoting recruitment of CAF [85]. It is worth noting that in solid tumors, the significant reduction in the blood vasculature accompanied by an increased deposition of collagen type I-enriched fibrotic tissue imposes a “barrier” effect that may further contribute to chemoresistance by decreasing drug delivery and availability at the tumor site [99].

### 3.4. Cancer Stem Cells

CSCs represent a peculiar sub-compartment of the tumor cell population crucially involved in tumor initiation and dissemination. CSCs are generally dormant or slowly cycling tumor cells with high tumorigenic potential, mostly localized as small clusters at the tumor-host interface, often along the external fibrous capsule [100,101]. CSCs are characterized by unlimited self-renewal capacity, hyperproliferation and the ability to reconstitute tumors by generating multiple tumor-derived cell types. CSCs also possess strong chemo- and radio-resistant properties and are responsible for disease recurrence after surgical or systemic interventions. Moreover, CSCs are, at least in part, responsible for metastatic dissemination of tumors, given their ability to escape from natural killer cell-dependent surveillance mechanisms [102]. CSCs have been identified in different types of epithelial cancers, including breast [103], pancreatic [104] and liver, both HCC [105] and CCA [106]. To date, the cell origin of CSC is controversial. It has been hypothesized that in liver tumors they might arise from dedifferentiation of hepatocytes or cholangiocytes, based on their phenotypic plasticity and high proliferative capacity, or from genetic/epigenetic alterations of hepatic progenitor cells (HPC). Indeed, the majority of liver malignancies, especially HCC, but also CCA, generally arise against a background of chronic liver disease, where both ductular metaplasia of hepatocytes and HPC activation are often intensely stimulated. HPCs are reactive cells with bipotential capabilities towards either hepatocyte or cholangiocyte lineages, located in a niche at the level of the canals of Hering or in the peribiliary glands. In experimental conditions, the HPC compartment is activated following extensive liver damage in order to replace the epithelial cell loss. Conversely, in human disease settings, HPCs are triggered even in mild forms of injury, without a replicative block of liver epithelial cells. In the presence of persistent, unresolving liver damage, proliferating HPCs can undergo genetic or epigenetic mutations due to the effects of mutagenic agents released in the inflammatory microenvironment, such as oxygen radicals or NO [107].

Notably, at variance with other epithelial cancers where the CSC population is usually minimally represented, in CCA, more than 30% of tumor cells express phenotypic markers of CSC [106], as outlined in Table 1. Among them, CD13, also known as aminopeptidase N, is a transmembrane glycoprotein expressed by circulating granulocytes and monocytes, but also by fibroblasts and by some epithelial cells lining the renal, intestinal and respiratory mucosa. In CCA, the expression of CD13 is typically found in mixed-iCCA subtypes [105], and as shown in HCC, it may protect cells from apoptosis and reduce DNA damage elicited by ROS induced by chemotherapeutic drugs, such as 5-FU [108]. Another CSC marker reported to be involved in chemoresistance is CD24, a membrane sialoglycoprotein aberrantly overexpressed in hematological malignancies, as well as in solid tumors, including CCA. In CCA, the expression of CD24 is associated with poor prognosis, reduced disease-free survival [110] and increased invasiveness, due to the activation of the ERK pathway and the upregulation of CXCR4, the cognate receptor for SDF-1/CXCL12 [111]. CD44 is the receptor for hyaluronic acid; in head and neck squamous cell carcinoma, hyaluronan enables CD44 to physically interact with the transcription factors Oct4, Sox2 and Nanog, which are critical determinants of embryonic cell differentiation. The resulting Oct4-Sox2-Nanog complex upregulates a number of pro-survival proteins (including XIAP) via microRNA (miR)-302, thereby leading to clonal formation and cisplatin resistance [122]. Laminin-332 is a matricellular peptide formed by three subunits, involved in cell adhesion and metastasization. In CCA, laminin-332 expression, in particular of its γ2-chain, is fundamental for maintaining theself-renewal abilities of CSCs and for inducing resistance to doxorubicin and sorafenib, an effect mediated by mTOR activation [120].

## 4. Main Signals Released within the TRS Promoting CCA Chemoresistance

**Figure 1:**
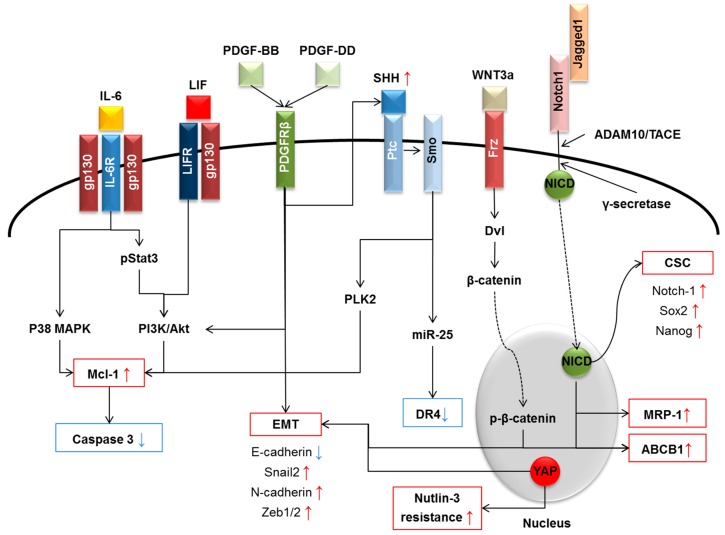
Molecular mechanisms of chemoresistance in CCA induced by autocrine and paracrine signals: IL-6/LIF, PDGF-BB/-DD, Hh, Wnt/β-catenin and Notch, released in the tumor microenvironment. Different effects on escape from apoptosis, modulation of transporter expression, induction of EMT properties and expansion of CSC compartment are illustrated. See the text for details. Abbreviations: ABCB1, ATP binding cassette subfamily B member 1; ADAM10, A disintegrin and metalloproteinase domain 10; CSC, cancer stem cells; DR4, death receptor 4; Dvl, disheveled; EMT, epithelial-mesenchymal transition; Frz, frizzled; gp130, glycoprotein 130; IL, interleukin; LIF, leukemia inhibitory factor; MAPK, mitogen-activated protein kinase; Mcl-1, myeloid cell leukemia 1; miR, micro-RNA; MRP1, multidrug resistance associated protein 1, NICD, intracellular domain of the notch protein; PDGF, platelet-derived growth factor; PI3K, phosphoinositide 3-kinase; PLK2, polo-like kinase 2; Ptc, patched; SHH, sonic hedgehog; Smo, smoothened; TACE, tumor necrosis factor, α, converting enzyme; YAP, yes associated protein 1. Red boxes and arrows: up-regulated proteins; blue boxes and arrows: down-regulated proteins.

In addition to stimulating the expansion of the CSC compartment, the multiple interactions established by the cellular components of the TRS with neoplastic bile ducts can directly promote the development of a chemoresistant phenotype, by activating specific intracellular pathways within the cancer cell. The extensive array of TRS-derived chemokines, growth factors and morphogens responsible for this pro-neoplastic effect are summarized in Figure 1. Some of these are considered more fully below.

### 4.1. Interleukin-6 (IL-6) Family

IL-6 can be considered the godfather of a large family of cytokines, which comprises, among others, IL-11, IL-27, Oncostatin M (OSM) and LIF. Members of the IL-6 family are major inflammatory mediators inducing chemoresistance in CCA, as well as one of the main molecular players of CCA tumorigenesis and progression. In CCA, IL-6 is produced not only by inflammatory cells and CAFs, but also by the malignant cholangiocytes themselves [123,124]. Autocrine and paracrine IL-6 facilitates CCA growth mainly by promoting survival of neoplastic cholangiocytes. For instance, overexpression of IL-6 in CCA cells significantly reduced the number of apoptotic cells following exposure to cytotoxic agents [124]. Similarly, CCA cells treated with a neutralizing anti-IL-6 antibody were more sensitized to TRAIL-mediated apoptosis [123]. The pro-survival effects of IL-6 mainly rely on the upregulation of the anti-apoptotic protein Mcl-1, via PI3K/Akt [123] and p38 mitogen-activated protein kinase (MAPK) pathways [124]. Interestingly, IL-6-mediated resistance to apoptosis in CCA cells was further enhanced by epigenetic silencing of the suppressor of cytokine signaling (SOCS)-3, a negative-feedback regulator of IL-6 signaling [2]. Stimulation of cell survival, however, is not the only mechanism by which IL-6 prompts tumor growth. In fact, previous studies unveiled the ability of IL-6 to encourage the proliferation of neoplastic cholangiocytes, through activation of p44/p42 and p38 MAPK [125].

Similar to IL-6, LIF can be secreted by numerous cell types, including stromal (fibroblasts, monocytes, macrophages, T cells) and epithelial (endometrial cells, hepatocytes, osteoblasts) cells [126,127]. Whereas data about possible involvement of IL-11, IL-27 and OSM in chemoresistance are hitherto lacking, interest in LIF is continuously increasing. LIF is a pleiotropic cytokine originally identified for its anti-proliferative action on M1 leukemic cells, but also able to regulate a number of biological activities, from blastocyst engraftment [128], to modulation of regulatory T cell activity [129] and bone metabolism [130], through to the maintenance of the multipotency of mesenchymal stem cells [131]. Moreover, LIF can either promote or inhibit cell proliferation and differentiation depending on the maturation stage of the cell type [126]. LIF binds to a heterodimeric receptor formed by two subunits, the ligand-specific low affinity subunit LIFR and the ubiquitous co-receptor gp130 [126,132,133]. Transduction of LIF signaling is associated with activation of variably integrated different pathways, including the Janus kinase (JAK)-signal transducer and activator of transcription (STAT)-3 and the PI3K/Akt pathways [126,127,134,135]. Overexpression of LIF and LIFR/gp130 was observed in CCA [136], as well as in other epithelial malignancies enriched in TRS, such as breast and prostate cancers [137,138]. 

Our recent studies show that cultured human CCA cells treated with LIF are protected from apoptosis induced by gemcitabine and cisplatin, an effect mediated by a STAT3- and MAPK-independent, PI3K/Akt-dependent Mcl-1 upregulation. Notably, other anti- or pro-apoptotic proteins, such as Bcl-2 and Bax, were unaffected [136]. Based on these observations, pharmacological strategies aimed at interfering with LIF signaling or its downstream effectors (Mcl-1 targeted by maritoclax) may offer new tools to increase CCA responsiveness to chemotherapeutic agents.

### 4.2. Platelet-Derived Growth Factor 

PDGF is a family of growth factors that act as potent mitogens for fibroblasts, smooth muscle cells and other cells of mesenchymal origin. PDGF family encompasses four isoforms of PDGF (from PDGF-A to -D) that specifically interact with two transmembrane tyrosine kinase receptors (PDGFR-α and -β). The ligands are all homodimers composed of polypeptide chains linked together by disulfide bridges, with the exception of one heterodimer (PDGF-AB), the physiological significance of which is uncertain [139,140]. While ligands may variably interact with both receptors in vitro, binding in vivo is more specific: PDGF-AA, -BB and -CC bind only PDGFR-α, while PDGF-BB and -DD bind only PDGFR-β [139]. In fibroblasts, mitogenic, motogenic and chemotactic effects of PDGF are regulated by the activation of several intracellular effectors, such as PI3K, phospholipase C (PLC), Ras (via the activation of Grb2/Sos), STAT [140], JNK and the Rho GTPases [85]. PDGF signaling is also known to play a crucial role in tumor progression. In HRas-transformed breast cancer cells, autocrine PDGFR signaling is aberrantly triggered during TGF-β-induced EMT, due to concomitant overexpression of both ligands and receptors. Sustained PDGF signaling is required for the maintenance of EMT (through activation of STAT1), as well as for the protection from apoptosis (through activation of PI3K) [141]. Besides being a critical mechanism of cell invasiveness, EMT also endows cancer cells with heightened resistance to apoptosis. For instance, EMT induction by PDGF-D represents a mechanism of chemoresistance in prostate cancer cells, where PDGF-D overexpression correlated with the downregulation of E-cadherin and increased expression of Snail2, N-cadherin, ZEB1, ZEB2 and vimentin [142]. Of further interest, in CCA, PDGF-BB, which is mainly secreted by CAF, enables cancer cells to become resistant to TRAIL-induced apoptosis. Anti-apoptotic effects of PDGF-BB rely on an intimate cross-talk with Hedgehog (Hh) signaling, based on the ability of PDGF-BB to stimulate the Hh coreceptor Smoothened (Smo), which leads to the activation of the transcription factor Gli and, in turn, to a reduced activation of caspase-3/7 [143]. In other cancer types, upregulation of PDGF-BB is reported even in neoplastic cells, due to hypoxia [95] or TGF-β stimulation [144]. A further effect on drug resistance exerted by the PDGF-BB/PDGFR-β axis that can be relevant in the TRS is the regulation of tumor interstitial pressure: in fact, PDGFR-β inhibition by imatinib increased uptake and thus enhanced the antitumor effects of cytotoxic drugs [139,144].

### 4.3. Wnt/β-Catenin

The complex Wnt/β-catenin morphogenetic pathway is highly conserved throughout evolution; it is involved in fetal development and in several fundamental cellular functions, dependent on the activation of either canonical or non-canonical Wnt signaling [145]. Originally discovered as part of the adherens junction complex, β-catenin was then identified as a transcriptional co-activator of Wnt target genes. By stimulating phosphorylation of β-catenin, the canonical pathway supervises a number of cellular processes underpinning hepatic reparative/regenerative mechanisms, including stemness activation and cell fate determination of progenitor cells [145]. On the other hand, the non-canonical pathway regulates cellular responses fundamental for morphogenesis, such as cell reshaping and motility, and planar cell polarity, which is necessary for ductal epithelia structuring [145]. The canonical pathway is activated by the binding of one of the several Wnt ligands to membrane receptors, these being frizzled (Fz) and its co-receptor low-density lipoprotein receptor related protein (LRP) 5/6. Normally, cytoplasmic β-catenin is segregated within a destruction complex, composed of the tumor suppressor Axin interacting with the adenomatous polyposis coli (APC), Wilms tumor gene on X chromosome (WTX) and other serine-threonine kinases (CK1α, -δ and -ε and GSK3α, -β) [146]. When Wnt stimulus is lacking, β-catenin is sequentially phosphorylated in different serine (Ser) and threonine (Thr) highly conserved residues (CK1 for Ser-45 and Thr-41, GSK3 for Ser-37 and Ser-33) [146]. Phosphorylation at Ser-33 and Ser-37 makes β-catenin targetable by the E3 ubiquitin ligase β-TrCP, eventually leading to its ubiquitination and proteasomal degradation [146]. Upon Wnt stimulation, β-catenin disruption is turned off, allowing its translocation into the nucleus, where it binds to the T cell factor/lymphoid enhancer factor (TCF/LEF) to activate Wnt target genes [146]. Recently, an alternative, non-canonical pathway of β-catenin activation has been described in cholangiocytes, induced by the cyclic adenosine monophosphate (cAMP)/protein kinase A (PKA) axis, which phosphorylates β-catenin at the Ser-552 and Ser-675 residues. This abnormal phosphorylation stabilizes β-catenin and inhibits its ubiquitination, allowing its nuclear translocation and transcriptional activation [147]. In CCA cells, aberrant activation of Wnt/β-catenin signaling is a relevant mechanism for drug resistance, as demonstrated both in vitro and in vivo. In particular, cultured CCA cells (QBC939) exposed to Wnt3a develop an increased resistance to several chemotherapeutic drugs, including 5-FU, cis-diammineplatinum, vincristine and mitomycin C. This effect was not mediated by the activation of the conventional pathways operating in chemoresistance (PI3K/Akt and NF-κB), but by the nuclear translocation of β-catenin, which is associated with the upregulation of ABCB1/P-glycoprotein [148,149]. Wnt signaling stimulation could be also obtained by co-culture of CCA cells with mesenchymal stem cells, which actually led to increased nuclear translocation of β-catenin, along with upregulation of Wnt target genes, namely MMP-2, cyclin D1 and c-Myc. Importantly, these phenotypic changes are functionally related to chemoresistance, both in vitro and in vivo [150]. Moreover, during cholestasis, Wnt7b and Wnt10a are also able to trigger cholangiocyte proliferation in a β-catenin-independent manner, acting through an autocrine loop [151].

### 4.4. Hippo Pathway

Also known as the Salvador/Warts/Hippo (SWH) pathway, the Hippo kinase cascade is, in common with Wnt/β-catenin, an evolutionarily-conserved pathway that controls several physiological and pathological cellular responses. It is critically involved in organ development, organ sizing, amplification of tissue-specific progenitor cells and tissue renewal, regeneration and repair. Not surprisingly, recent data strongly support dysregulation of the Hippo pathway as significantly involved in cancer progression and metastatic dissemination [152,153]. In Hippo signaling, mammalian sterile 20-like kinase (Mst)-1/2 (the human orthologues of the *Drosophila* Hippo protein) binds to the Salvador family WW domain containing protein (SAV)-1 to form a complex that phosphorylates and activates large tumor suppressor kinase (LATS)-1/2. In turn, LATS1/2 maintains in a phosphorylated state the two main downstream effectors of the Hippo pathway, the transcriptional co-activator yes-associated protein (YAP) and its paralog, transcriptional co-activator with PDZ-binding motif (TAZ). While YAP/TAZ are phosphorylated, they are maintained as integral components of the β-catenin destruction complex [154,155]. Once dephosphorylated, YAP/TAZ translocate into the nucleus to activate several transcription factors, such as the TEA-domain (TEAD)1–4 [154,155], dictating the expression of several mitogenic genes, among which are connective tissue growth factor (CTGF) (which is relevant in liver fibrosis), ankyrin repeat domain 1 and cysteine-rich angiogenic inducer 61 [156]. YAP plays a broad pro-oncogenic role, from regulation of the balance between proliferation and apoptosis, to induction of motility/EMT, and its perturbation has been reported in liver, skin, breast and lung carcinomas, among others [157]. In human CCA cells, forced expression of a constitutively active form of YAP led to decreased expression of TRAIL, resulting in enhanced resistance to Nutlin-3, a chemotherapeutic drug preventing the interaction between p53 and its inhibitor MDM2. In CCA, both in vitro and in vivo, YAP activation also resulted in increased tumor cell proliferation and angiogenesis [158].

### 4.5. Notch

Notch is a developmental signal involved in cell fate determination and tissue morphogenesis, acting through a mechanism of mutual cell contact, which exerts opposing effects of ‘lateral inhibition’ and ‘lateral induction’. In adults, Notch signaling regulates self-renewal and differentiation of organ-specific stem cells, a fundamental mechanism in liver repair and regeneration. Cell-cell contacts are required for Notch signaling activation because both ligands (Jagged 1 and 2 and Delta 1, 2 and 4) and receptors (Notch 1–4) are bound to the cell surface. Following physical interaction between “sending” and “receiving” cells, the Notch intracellular domain (NICD) undergoes a sequential proteolitic cleavage by ADAM10/TACE metalloprotease first and then by γ-secretase. During development of the intrahepatic biliary epithelium, NICD enters the nucleus and links to a specific DNA-binding partner, called recombination signal binding protein immunoglobulin kappa J (RBP-Jk), in order to stimulate expression of Notch target genes. Among them, hepatocyte nuclear factor (HNF)-1β, sex determining region Y-box (Sox)-9, hairy and enhancer of split (Hes)-1 and -5, hairy/enhancer-of-split-related with the YRPW motif (Hey)-1 and -2 are relevant for biliary cell specification [159]. Dysregulation of Notch signaling, affecting both ligands and receptors, has been frequently reported in the pathogenesis of several epithelial malignancies, including cervical, craniofacial, renal, lung, pancreatic, ovarian and gastric carcinomas, as well as HCC and CCA [160,161]. A growing body of data pinpoints the peculiar ability of Notch to transform HPCs. In more than 30% of HCC, neoplastic hepatocytes showed nuclear expression of Notch 1 and 3, which was associated with both over-activation of Notch target genes and expression of stem cell signatures, likely supporting the role of Notch in promoting the expansion of the CSC niche [162,163]. Furthermore, persistent activation of Notch in hepatocytes, coupled with stimulation of Akt signaling, converted normal hepatocytes into cholangiocytes, eventually behaving as precursors of a rapidly progressing, highly invasive iCCA [164]. Similarly, in a transgenic mouse model, overactivation of NICD within HPCs led to intrahepatic cholangiocarcinogenesis, due to persistent activation of cyclin E1, which ultimately induced DNA double-strand breaks. Of note, blocking of γ-secretase activity downregulated cyclin E1 expression, an effect associated with the induction of cancer cell apoptosis and subsequent tumor remission [165]. Furthermore, down-modulation of Notch 1 in cultured iCCA cells (RBE and HCCC-9810) decreased the expression of two efflux pumps, namely ABCB1/P-glycoprotein and multidrug resistance-associated protein 1 (MRP1), making these cells more sensitive to toxicity induced by 5-FU [166].

### 4.6. Hedgehog

Hedgehog (Hh) is a pathway promoting cooperative morphogenesis of different tissues and organs and, in fetal liver, to differentiation of both hepatocytes and cholangiocytes from hepatoblasts, in concert with Notch [167]. In physiological conditions, this pathway is repressed by the inhibitory binding of the canonical receptor Patched (Ptc) to the G protein-coupled receptor-like Smo. When Ptc is bound by its specific ligands, (i.e., Sonic (Shh), Desert (Dhh) and Indian (Ihh)), the inhibitory link between the two receptors is lost, allowing Smo phosphorylation/activation. This leads to disruption of the cytoplasmic complex composed of suppressive fused (Su(Fu)), kinesin family member (Kif)-7 and Gli proteins 1/2/3. Once released from this complex, Gli proteins are processed into transcriptional activators, which enter the nucleus to activate the transcription of Hh-related genes, such as Gli1, Ptc, Cyclin D, Cyclin E and c-Myc [145]. Features consistent with Hh activation have recently been described in nearly 50% of CCA [168], where they correlate with an increased survival of neoplastic cholangiocytes [167], potentially underlying the development of chemoresistance. In CCA cells (KMCH, HuCCT-1, Mz-Cha-1), Hh signaling was constitutively active and suppressed the expression of DR4, thereby increasing cancer cell resistance to TRAIL-induced apoptosis, an effect blunted by the Smo inhibitor cyclopamine [169]. In particular, the Hh-mediated DR4 downregulation relied on both the functional activation of Gli3, which directly repressed DR4 promoter function [169], and the transcriptional activation of miR-25, which inhibited the translation of DR4 mRNA [170]. In addition, autocrine Hh signaling (especially Gli1 and Gli2) further counteracted CCA cell apoptosis by upregulating the expression of the cell division regulator polo-like kinase (PLK)-2, which prevented Mcl-1 from proteasomal degradation [171]. Consistent with these in vitro findings, growth of human CCA cells xenografted in nude mice was partly hampered by different strategies of Hh blockade, namely BMS-833923, a small-molecule Smo inhibitor [168], cyclopamine [168,172] or BI 6727, a PLK inhibitor [171].

## 5. Concluding Remarks and Future Directions

The increasing incidence of CCA in Western countries is placing a pronounced burden on health providers. Owing to the current paucity of effective therapies, CCA remains a devastating disease, which still carries an extremely poor prognosis. Curative surgery, including resection or liver transplant, represents a therapeutic option only if the tumor is diagnosed early enough and is a viable option for only a minority of patients. In most patients, CCA is recognized only at an advanced stage, but chemotherapy, which conventionally has been ruled out, is still currently yielding limited beneficial results, due in part at least to the strong chemoresistance of this tumor. The highly aggressive nature of CCA and its rapid progression are supported by the rich stromal reaction that typically develops in close association with the neoplastic epithelial growth. Indeed, within the desmoplastic microenvironment, a multitude of autocrine and paracrine signals are released, facilitating close interactions between the stromal and cellular compartments. Many of the soluble factors, including cyto/chemokines (e.g., IL-6), growth factors (e.g., PDGF) and morphogens (e.g., Wnt/β-catenin, Hippo, Notch and Hh), are also relevant for the development of a chemoresistant phenotype, either by providing CCA cells with tools to escape from drug-induced apoptosis or by expanding the CSC compartment. As previously outlined, by dissecting the intimate nature of these molecular mechanisms (Figure 1), new therapeutic strategies that may circumvent chemoresistance are emerging. For instance, the IL-6/LIF effector Mcl-1 (by maritoclax), PDGFR-β (by imatinib), Wnt/β-catenin signaling (by XAV939, which promotes β-catenin degradation by stabilizing axin) and Hh signaling (by cyclopamine) are all putative targets amenable of intervention to sensitize CCA cells to chemotherapeutic drugs. These molecular targets may provide novel approaches to be used in combination, following the example of cisplatin and gemcitabine, as originally proposed by Valle [10]. Among them, several drugs and small molecules are under phase I/II investigation in different epithelial cancers, such as Dactolisib (PI3K inhibitor), Maritoclax (Mcl-1 inhibitor) and Tariquidar (MDR1 inhibitor), but to date, none of them is under consideration for CCA treatment.

Additional inputs are awaited from studies further unraveling the inflammatory and the immunological responses sustaining CCA chemoresistance. One of the main “grey” areas deserving more attention, is the complex role played by tumor-associated macrophages. In addition to M2 macrophages, M1 macrophages in cooperation with other inflammatory cell types (neutrophils, NK cells) could also be actively involved in desensitizing neoplastic cholangiocytes to cytotoxic drugs and stimulating CSC expansion. Moreover, an emerging therapeutic strategy is the attractive capability to interfere with the CSC compartment. CSC may be regarded as a potential “Achilles’ heel” for different types of tumors [173], because they bear several surface antigens amenable to immunotherapy intervention. For instance, polymer nanoparticles impregnated with paclitaxel specifically targeting CD133 have been recently used to treat colorectal adenocarcinoma cells in vitro with promising results [174]. Other surface markers under investigation as potential opportunities of anti-CSC therapies are CD44 and CD47, druggable with specific monoclonal antibodies [175,176].

It is hoped that expansion of knowledge in the areas reviewed will ultimately widen the subset of CCA patients eligible for radical treatments in the near future, thus alleviating the disease burden of this malignancy.

## Figures and Tables

**Table 1:** Biomarkers of cancer stem cells (CSC) expressed in cholangiocarcinoma (CCA).

Biomarker	Molecular Identity	Biological Significance and Relevance	Ref.
**CD13**	Transmembrane glycoprotein expressed by granulocytes, monocytes, fibroblasts and some epithelial cells	Escape from drug-induced apoptosis, reported in HCC	[106,108]
**CD24**	Membrane sialoglycoprotein overexpressed in hematological and epithelial malignancies	Increased cell invasiveness, marker of poor outcome in CCA	[109,110,111]
**CD44**	Transmembrane hyaluronic acid binding glycoprotein overexpressed in several epithelial cancers	Increased tumorigenicity by synergizing with other peptides	[112,113]
**CD90**	Phosphatidyl-bound cell surface glycoprotein, expressed by mesenchymal stem cells and by CSC in HCC and CCA	Unknown in CCA, proposed as CSC marker	[106,114,115]
**CD133**	Transmembrane glycoprotein expressed by hematopoietic stem cells, adult progenitor cells and in fetal liver	Possible marker of poor outcome in CCA	[116,117,118]
**EpCAM**	Adhesion molecule involved in cell-cell interactions, overexpressed in several tumors	Unknown in CCA, proposed as CSC marker	[119]
**Laminin-332**	Matricellular peptide involved in cell adhesion and metastasization	Preserved stemness of CSC and induced resistance to doxorubicin and sorafenib, in CCA	[120]
**LGR5**	G protein-coupled receptor expressed by CSC in CCA	Unknown in CCA, proposed as CSC marker	[106]
**Nanog**	Transcription factor regulating developmental features	Involved in self-renewal and differentiation of embryonic stem cells, used as CSC marker	[109,121]
**Sox2**	Transcription factor regulating developmental features	Involved in stem cell differentiation, correlation with increased lymphatic metastasization and poor outcome in CCA	[113]

EpCAM, epithelial cell adhesion molecule; LGR5, leucine rich repeat containing G protein-coupled receptor 5; Sox2, sex-determining region Y-box 2.

## References

[1] Blechacz B., Gores G.J. (2008). Cholangiocarcinoma: Advances in pathogenesis, diagnosis, and treatment. Hepatology.

[2] Gatto M., Bragazzi M.C., Semeraro R., Napoli C., Gentile R., Torrice A., Gaudio E., Alvaro D. (2010). Cholangiocarcinoma: Update and future perspectives. Dig. Liver Dis..

[3] Floreani A., Lisiero M., Baldovin T., Baldo V. (2013). Epidemiological aspects of biliary tree tumors in a region of northern Italy: Emerging trends and sex-based differences. Eur. J. Gastroenterol. Hepatol..

[4] Bragazzi M., Cardinale V., Carpino G., Venere R., Semeraro R., Gentile R., Gaudio E., Alvaro D. (2011). Cholangiocarcinoma: Epidemiology and risk factors. Transl. Gastrointest. Cancer.

[5] Wolpin B.M., Mayer R.J. (2010). A step forward in the treatment of advanced biliary tract cancer. N. Engl. J. Med..

[6] Razumilava N., Gores G.J. (2013). Classification, diagnosis, and management of cholangiocarcinoma. Clin. Gastroenterol. Hepatol..

[7] Bridgewater J., Galle P.R., Khan S.A., Llovet J.M., Park J.W., Patel T., Pawlik T.M., Gores G.J. (2014). Guidelines for the diagnosis and management of intrahepatic cholangiocarcinoma. J. Hepatol..

[8] Zabron A., Edwards R.J., Khan S.A. (2013). The challenge of cholangiocarcinoma: Dissecting the molecular mechanisms of an insidious cancer. Dis. Model. Mech..

[9] Marin J.J., Lozano E., Briz O., Al-Abdulla R., Serrano M.A., Macias R.I. (2015). Molecular Bases of Chemoresistance In Cholangiocarcinoma. Curr. Drug Targets.

[10] Valle J., Wasan H., Palmer D.H., Cunningham D., Anthoney A., Maraveyas A., Madhusudan S., Iveson T., Hughes S., Pereira S.P. (2010). ABC-02 Trial Investigators. Cisplatin plus gemcitabine versus gemcitabine for biliary tract cancer. N. Engl. J. Med..

[11] Pan S.T., Li Z.L., He Z.X., Qiu J.X., Zhou S.F. (2016). Molecular mechanisms for tumour resistance to chemotherapy. Clin. Exp. Pharmacol. Physiol..

[12] Banales J.M., Cardinale V., Carpino G., Marzioni M., Andersen J.B., Invernizzi P., Lind G.E., Folseraas T., Forbes S.J., Fouassier L. (2016). Expert consensus document: Cholangiocarcinoma: Current knowledge and future perspectives consensus statement from the European Network for the Study of Cholangiocarcinoma (ENS-CCA). Nat. Rev. Gastroenterol. Hepatol..

[13] Strazzabosco M., Fabris L., Spirli C. (2005). Pathophysiology of cholangiopathies. J. Clin. Gastroenterol..

[14] Franke R.M., Scherkenbach L.A., Sparreboom A. (2009). Pharmacogenetics of the organic anion transporting polypeptide 1A2. Pharmacogenomics.

[15] Wlcek K., Svoboda M., Riha J., Zakaria S., Olszewski U., Dvorak Z., Sellner F., Ellinger I., Jäger W., Thalhammer T. (2011). The analysis of organic anion transporting polypeptide (OATP) mRNA and proteinpatterns in primary and metastatic liver cancer. Cancer Biol. Ther..

[16] Herraez E., Lozano E., Macias R.I., Vaquero J., Bujanda L., Banales J.M., Marin J.J., Briz O. (2013). Expression of SLC22A1 variants may affect the response of hepatocellular carcinoma and cholangiocarcinoma to sorafenib. Hepatology.

[17] Marin J.J. (2012). Plasma membrane transporters in modern liver pharmacology. Scientifica.

[18] Namwat N., Amimanan P., Loilome W., Jearanaikoon P., Sripa B., Bhudhisawasdi V., Tassaneeyakul W. (2008). Characterization of 5-fluorouracil resistant cholangiocarcinoma cell lines. Chemotherapy.

[19] Ferreira R.J., dos Santos D.J., Ferreira M.J. (2015). P-glycoprotein and membrane roles in multidrug resistance. Future Med. Chem..

[20] Eaton J.E., Talwalkar J.A., Lazaridis K.N., Gores G.J., Lindor K.D. (2013). Pathogenesis of primary sclerosing cholangitis and advances in diagnosis and management. Gastroenterology.

[21] Perez J., Bardin C., Rigal C., Anthony B., Rousseau R., Dutour A. (2011). Anti-MDR1 siRNA restores chemosensitivity in chemoresistant breast carcinoma and osteosarcoma cell lines. Anticancer Res..

[22] Hahnvajanawong C., Chaiyagool J., Seubwai W., Bhudhisawasdi V., Namwat N., Khuntikeo N., Sripa B., Pugkhem A., Tassaneeyakul W. (2012). Orotate phosphoribosyl transferase mRNA expression and the response of cholangiocarcinoma to 5-fluorouracil. World J. Gastroenterol..

[23] Nakajima T., Takayama T., Miyanishi K., Nobuoka A., Hayashi T., Abe T., Kato J., Sakon K., Naniwa Y., Tanabe H. (2003). Reversal of multiple drug resistance in cholangiocarcinoma by the glutathione S-transferase-pi-specific inhibitor *O*1-hexadecyl-γ-glutamyl-*S*-benzylcysteinyl-d-phenylglycine ethylester. J. Pharmacol. Exp. Ther..

[24] Jansen W.J., Kolfschoten G.M., Erkelens C.A., Van Ark-Otte J., Pinedo H.M., Boven E. (1997). Anti-tumor activity of CPT-11 in experimental human ovarian cancer and human soft-tissue sarcoma. Int. J. Cancer.

[25] Boyer J., McLean E.G., Aroori S., Wilson P., McCulla A., Carey P.D., Longley D.B., Johnston P.G. (2004). Characterization of p53 wild-type and null isogenic colorectal cancer cell lines resistant to 5-fluorouracil, oxaliplatin, and irinotecan. Clin. Cancer Res..

[26] Alvaro D., Barbaro B., Franchitto A., Onori P., Glaser S.S., Alpini G., Francis H., Marucci L., Sterpetti P., Ginanni-Corradini S. (2006). Estrogens and insulin-like growth factor 1 modulate neoplastic cell growth in human cholangiocarcinoma. Am. J. Pathol..

[27] Sampson L.K., Vickers S.M., Ying W., Phillips J.O. (1997). Tamoxifen-mediated growth inhibition of human cholangiocarcinoma. Cancer Res..

[28] Marzioni M., Torrice A., Saccomanno S., Rychlicki C., Agostinelli L., Pierantonelli I., Rhönnstad P., Trozzi L., Apelqvist T., Gentile R. (2012). An oestrogen receptor β-selective agonist exerts anti-neoplastic effects in experimental intrahepatic cholangiocarcinoma. Dig. Liver Dis..

[29] Hector S., Bolanowska-Higdon W., Zdanowicz J., Hitt S., Pendyala L. (2001). In vitro studies on the mechanisms of oxaliplatin resistance. Cancer Chemother. Pharmacol..

[30] Metzger R., Leichman C.G., Danenberg K.D., Danenberg P.V., Lenz H.J., Hayashi K., Groshen S., Salonga D., Cohen H., Laine L. (1998). ERCC1 mRNA levels complement thymidylate synthase mRNA levels in predicting response and survival for gastric cancer patients receiving combination cisplatin and fluorouracil chemotherapy. J. Clin. Oncol..

[31] Hwang I.G., Jang J.S., Do J.H., Kang J.H., Lee G.W., Oh S.Y., Kwon H.C., Jun H.J., Lim H.Y., Lee S. (2011). Different relation between ERCC1 overexpression and treatment outcomes of two platinum agents in advanced biliary tract adenocarcinoma patients. Cancer Chemother. Pharmacol..

[32] Selvakumaran M., Pisarcik D.A., Bao R., Yeung A.T., Hamilton T.C. (2003). Enhanced cisplatin cytotoxicity by disturbing the nucleotide excision repair pathway in ovarian cancer cell lines. Cancer Res..

[33] Asakawa H., Koizumi H., Koike A., Takahashi M., Wu W., Iwase H., Fukuda M., Ohta T. (2010). Prediction of breast cancer sensitivity to neoadjuvant chemotherapy based on status of DNA damage repair proteins. Breast Cancer Res..

[34] Obama K., Satoh S., Hamamoto R., Sakai Y., Nakamura Y., Furukawa Y. (2008). Enhanced expression of RAD51 associating protein-1 is involved in the growth of intrahepatic cholangiocarcinoma cells. Clin. Cancer Res..

[35] Fink D., Aebi S., Howell S.B. (1998). The role of DNA mismatch repair in drug resistance. Clin. Cancer Res..

[36] Chaney S.G., Campbell S.L., Temple B., Bassett E., Wu Y., Faldu M. (2004). Protein interactions with platinum-DNA adducts: From structure to function. J. Inorg. Biochem..

[37] Vogler M. (2014). Targeting BCL2-Proteins for the Treatment of Solid Tumours. Adv. Med..

[38] Harnois D.M., Que F.G., Celli A., LaRusso N.F., Gores G.J. (1997). Bcl-2 is overexpressed and alters the threshold for apoptosis in a cholangiocarcinoma cell line. Hepatology.

[39] Fabris L., Strazzabosco M., Crosby H.A., Ballardini G., Hubscher S.G., Kelly D.A., Neuberger J.M., Strain A.J., Joplin R. (2000). Characterization and isolation of ductular cells coexpressing neural cell adhesion molecule and Bcl-2 from primary cholangiopathies and ductal plate malformations. Am. J. Pathol..

[40] Minagawa N., Kruglov E.A., Dranoff J.A., Robert M.E., Gores G.J., Nathanson M.H. (2005). The anti-apoptotic protein Mcl-1 inhibits mitochondrial Ca^2+^ signals. J. Biol. Chem..

[41] Li R., Cheng C., Balasis M.E., Liu Y., Garner T.P., Daniel K.G., Li J., Qin Y., Gavathiotis E., Sebti S.M. (2015). Design, synthesis and evaluation of marinopyrrole derivatives as selective inhibitors of Mcl-1 binding to pro-apoptotic Bim and dual Mcl-1/Bcl-xL inhibitors. Eur. J. Med. Chem..

[42] Ma J., Zhao Z., Wu K., Xu Z., Liu K. (2016). MCL-1 is the key target of adjuvant chemotherapy to reverse the cisplatin-resistance in NSCLC. Gene.

[43] Liao M., Zhao J., Wang T., Duan J., Zhang Y., Deng X. (2011). Role of bile salt in regulating Mcl-1 phosphorylation and chemoresistance in hepatocellular carcinoma cells. Mol. Cancer.

[44] Rampino N., Yamamoto H., Ionov Y., Li Y., Sawai H., Reed J.C., Perucho M. (1997). Somatic frameshift mutations in the *BAX* gene in colon cancers of the microsatellite mutator phenotype. Science.

[45] Kymionis G.D., Dimitrakakis C.E., Konstadoulakis M.M., Arzimanoglou I., Leandros E., Chalkiadakis G., Keramopoulos A., Michalas S. (2001). Can expression of apoptosis genes, *bcl-2* and *bax*, predict survival and responsiveness to chemotherapy in node-negative breast cancer patients?. J. Surg. Res..

[46] Sjöström J., Blomqvist C., von Boguslawski K., Bengtsson N.O., Mjaaland I., Malmström P., Ostenstadt B., Wist E., Valvere V., Takayama S. (2002). The predictive value of bcl-2, bax, bcl-xL, bag-1, fas, and fasL for chemotherapy response in advanced breast cancer. Clin. Cancer Res..

[47] Paradiso A., Simone G., Lena M.D., Leone B., Vallejo C., Lacava J., Dellapasqua S., Daidone M.G., Costa A. (2001). Expression of apoptosis-related markers and clinical outcome in patients with advanced colorectal cancer. Br. J. Cancer.

[48] Yoon H., Min J.K., Lee J.W., Kim D.G., Hong H.J. (2011). Acquisition of chemoresistance in intrahepatic cholangiocarcinoma cells by activation of AKT and extracellular signal-regulated kinase (ERK)1/2. Biochem. Biophys. Res. Commun..

[49] Bunz F., Hwang P.M., Torrance C., Waldman T., Zhang Y., Dillehay L., Williams J., Lengauer C., Kinzler K.W., Vogelstein B. (1999). Disruption of p53 in human cancer cells alters the responses to therapeutic agents. J. Clin. Investig..

[50] Khan S.A., Thomas H.C., Toledano M.B., Cox I.J., Taylor-Robinson S.D. (2005). p53 Mutations in human cholangiocarcinoma: A review. Liver Int..

[51] Cheng J.Q., Jiang X., Fraser M., Li M., Dan H.C., Sun M., Tsang B.K. (2002). Role of X-linked inhibitor of apoptosis protein in chemoresistance in ovarian cancer: Possible involvement of the phosphoinositide-3 kinase/Akt pathway. Drug Resist. Updat..

[52] Tamm I., Wang Y., Sausville E., Scudiero D.A., Vigna N., Oltersdorf T., Reed J.C. (1998). IAP-family protein survivin inhibits caspase activity and apoptosis induced by Fas (CD95), Bax, caspases, and anticancer drugs. Cancer Res..

[53] Zaffaroni N., Pennati M., Colella G., Perego P., Supino R., Gatti L., Pilotti S., Zunino F., Daidone M.G. (2002). Expression of the anti-apoptotic gene survivin correlates with taxol resistance in human ovarian cancer. Cell. Mol. Life Sci..

[54] Kato J., Kuwabara Y., Mitani M., Shinoda N., Sato A., Toyama T., Mitsui A., Nishiwaki T., Moriyama S., Kudo J. (2001). Expression of survivin in esophageal cancer: Correlation with the prognosis and response to chemotherapy. Int. J. Cancer.

[55] Martinez-Becerra P., Vaquero J., Romero M.R., Lozano E., Anadon C., Macias R.I., Serrano M.A., Grañé-Boladeras N., Muñoz-Bellvis L., Alvarez L. (2012). No correlation between the expression of FXR and genes involved in multidrug resistance phenotype of primary liver tumors. Mol. Pharm..

[56] Wehrkamp C.J., Gutwein A.R., Natarajan S.K., Phillippi M.A., Mott J.L. (2014). XIAP antagonist embelin inhibited proliferation of cholangiocarcinoma cells. PLoS ONE.

[57] Ueno Y., Ishii M., Yahagi K., Mano Y., Kisara N., Nakamura N., Shimosegawa T., Toyota T., Nagata S. (2000). Fas-mediated cholangiopathy in the murine model of graft versus host disease. Hepatology.

[58] Alvaro D., Alpini G., Onori P., Perego L., Svegliati-Baroni G., Franchitto A., Baiocchi L., Glaser S.S., Le Sage G., Folli F. (2000). Estrogens stimulate proliferation of intrahepatic biliary epithelium in rats. Gastroenterology.

[59] Takeda K., Kojima Y., Ikejima K., Harada K., Yamashina S., Okumura K., Aoyama T., Frese S., Ikeda H., Haynes N.M. (2008). Death receptor 5 mediated-apoptosis contributes to cholestatic liver disease. Proc. Natl. Acad. Sci. USA.

[60] Micheau O., Shirley S., Dufour F. (2013). Death receptors as targets in cancer. Br. J. Pharmacol..

[61] Jin Z., McDonald E.R., Dicker D.T., El-Deiry W.S. (2004). Deficient tumor necrosis factor-related apoptosis-inducing ligand (TRAIL) death receptor transport to the cell surface in human colon cancer cells selected for resistance to TRAIL-induced apoptosis. J. Biol. Chem..

[62] Wise J.F., Berkova Z., Mathur R., Zhu H., Braun F.K., Tao R.H., Sabichi A.L., Ao X., Maeng H., Samaniego F. (2013). Nucleolin inhibits Fas ligand binding and suppresses Fas-mediated apoptosis in vivo via a surface nucleolin-Fas complex. Blood.

[63] Fulda S. (2014). Tumor-necrosis-factor-related apoptosis-inducing ligand (TRAIL). Adv. Exp. Med. Biol..

[64] Longley D.B., Wilson T.R., McEwan M., Allen W.L., McDermott U., Galligan L., Johnston P.G. (2006). c-FLIP inhibits chemotherapy-induced colorectal cancer cell death. Oncogene.

[65] Okano H., Shiraki K., Inoue H., Kawakita T., Yamanaka T., Deguchi M., Sugimoto K., Sakai T., Ohmori S., Fujikawa K. (2003). Cellular FLICE/caspase-8-inhibitory protein as a principal regulator of cell death and survival in human hepatocellular carcinoma. Lab. Investig..

[66] Fernandez T.F., Samal A.B., Bedwell G.J., Chen Y., Saad J.S. (2013). Structural and biophysical characterization of the interactions between the death domain of Fas receptor and calmodulin. J. Biol. Chem..

[67] Lin A., Karin M. (2003). NF-kappaB in cancer: A marked target. Semin. Cancer Biol..

[68] Kato T., Duffey D.C., Ondrey F.G., Dong G., Chen Z., Cook J.A., Mitchell J.B., Van Waes C. (2000). Cisplatin and radiation sensitivity in human head and neck squamous carcinomas are independently modulated by glutathione and transcription factor NF-κB. Head Neck.

[69] Arlt A., Gehrz A., Müerköster S., Vorndamm J., Kruse M.L., Fölsch U.R., Schäfer H. (2003). Role of NF-κB and Akt/PI3K in the resistance of pancreatic carcinoma cell lines against gemcitabine-induced cell death. Oncogene.

[70] Gores G.J. (2003). Cholangiocarcinoma: Current concepts and insights. Hepatology.

[71] Cadamuro M., Morton S.D., Strazzabosco M., Fabris L. (2013). Unveiling the Role of Tumor Reactive Stroma in Cholangiocarcinoma: An Opportunity for New Therapeutic Strategies. Transl. Gastrointest. Cancer.

[72] Kalluri R., Zeisberg M. (2006). Fibroblasts in cancer. Nat. Rev. Cancer.

[73] Duluc D., Delneste Y., Tan F., Moles M.P., Grimaud L., Lenoir J., Preisser L., Anegon I., Catala L., Ifrah N. (2007). Tumor-associated leukemia inhibitory factor and IL-6 skew monocyte differentiation into tumor-associated macrophage-like cells. Blood.

[74] Dvorak H.F. (1986). Tumors: Wounds that do not heal. Similarities between tumor stroma generation and wound healing. N. Engl. J. Med..

[75] Schober M., Jesenofsky R., Faissner R., Weidenauer C., Hagmann W., Michl P., Heuchel R.L., Haas S.L., Löhr J.M. (2014). Desmoplasia and chemoresistance in pancreatic cancer. Cancers.

[76] Velaei K., Samadi N., Barazvan B., Soleimani Rad J. (2016). Tumor microenvironment-mediated chemoresistance in breast cancer. Breast.

[77] Jayson G.C., Kohn E.C., Kitchener H.C., Ledermann J.A. (2014). Ovarian cancer. Lancet.

[78] Zhang W., Meng Y., Liu N., Wen X.F., Yang T. (2015). Insights into Chemoresistance of Prostate Cancer. Int. J. Biol. Sci..

[79] Goubran H.A., Kotb R.R., Stakiw J., Emara M.E., Burnouf T. (2014). Regulation of tumor growth and metastasis: The role of tumor microenvironment. Cancer Growth Metastasis.

[80] Yamaguchi H., Sakai R. (2015). Direct Interaction between Carcinoma Cells and Cancer Associated Fibroblasts for the Regulation of Cancer Invasion. Cancers.

[81] McCarroll J.A., Naim S., Sharbeen G., Russia N., Lee J., Kavallaris M., Goldstein D., Phillips P.A. (2014). Role of pancreatic stellate cells in chemoresistance in pancreatic cancer. Front. Physiol..

[82] Chi J.Y., Hsiao Y.W., Li C.F., Lo Y.C., Lin Z.Y., Hong J.Y., Liu Y.M., Han X., Wang S.M., Chen B.K. (2015). Targeting chemotherapy-induced PTX3 in tumor stroma to prevent the progression of drug-resistant cancers. Oncotarget.

[83] Chu A.S., Diaz R., Hui J.J., Yanger K., Zong Y., Alpini G., Stanger B.Z., Wells R.G. (2011). Lineage tracing demonstrates no evidence of cholangiocyte epithelial-to-mesenchymal transition in murine models of hepatic fibrosis. Hepatology.

[84] Kisseleva T., Brenner D.A. (2011). Is it the end of the line for the EMT?. Hepatology.

[85] Cadamuro M., Nardo G., Indraccolo S., Dall’olmo L., Sambado L., Moserle L., Franceschet I., Colledan M., Massani M., Stecca T. (2013). Platelet-derived growth factor-D and Rho GTPases regulate recruitment of cancer-associated fibroblasts in cholangiocarcinoma. Hepatology.

[86] Fabris L., Strazzabosco M. (2011). Epithelial-mesenchymal interactions in biliary diseases. Semin. Liver Dis..

[87] Clapéron A., Mergey M., Aoudjehane L., Ho-Bouldoires T.H., Wendum D., Prignon A., Merabtene F., Firrincieli D., Desbois-Mouthon C., Scatton O. (2013). Hepatic myofibroblasts promote the progression of human cholangiocarcinoma through activation of epidermal growth factor receptor. Hepatology.

[88] Rizvi S., Gores G.J. (2013). Pathogenesis, diagnosis, and management of cholangiocarcinoma. Gastroenterology.

[89] Utispan K., Sonongbua J., Thuwajit P., Chau-In S., Pairojkul C., Wongkham S., Thuwajit C. (2012). Periostin activates integrin α5β1 through a PI3K/AKT-dependent pathway in invasion of cholangiocarcinoma. Int. J. Oncol..

[90] Sirica A.E., Almenara J.A., Li C. (2014). Periostin in intrahepatic cholangiocarcinoma: Pathobiological insights and clinical implications. Exp. Mol. Pathol..

[91] Hasita H., Komohara Y., Okabe H., Masuda T., Ohnishi K., Lei X.F., Beppu T., Baba H., Takeya M. (2010). Significance of alternatively activated macrophages in patients with intrahepatic cholangiocarcinoma. Cancer Sci..

[92] Raggi C., Correnti M., Sica A., Andersen J.B., Cardinale V., Alvaro D., Chiorino G., Forti E., Glaser S., Alpini G. (2017). Cholangiocarcinoma stem-like subset shapes tumor-initiating niche by educating associated macrophages. J. Hepatol..

[93] Locatelli L., Cadamuro M., Spirlì C., Fiorotto R., Lecchi S., Morell C.M., Popov Y., Scirpo R., de Matteis M., Amenduni M. (2016). Macrophage recruitment by fibrocystin-defective biliary epithelial cells promotes portal fibrosis in congenital hepatic fibrosis. Hepatology.

[94] Lewis C.E., Pollard J.W. (2006). Distinct role of macrophages in different tumor microenvironments. Cancer Res..

[95] Schito L., Rey S., Tafani M., Zhang H., Wong C.C., Russo A., Russo M.A., Semenza G.L. (2012). Hypoxia-inducible factor 1-dependent expression of platelet-derived growth factor B promotes lymphatic metastasis of hypoxic breast cancer cells. Proc. Natl. Acad. Sci. USA.

[96] Karnezis T., Shayan R., Caesar C., Roufail S., Harris N.C., Ardipradja K., Zhang Y.F., Williams S.P., Farnsworth R.H., Chai M.G. (2012). VEGF-D promotes tumor metastasis by regulating prostaglandins produced by the collecting lymphatic endothelium. Cancer Cell.

[97] Cheng H.T., Hung W.C. (2013). Inhibition of proliferation, sprouting, tube formation and Tie2 signaling of lymphatic endothelial cells by the histone deacetylase inhibitor SAHA. Oncol. Rep..

[98] Morine Y., Shimada M., Utsunomiya T., Imura S., Ikemoto T., Mori H., Hanaoka J., Kanamoto M., Iwahashi S., Miyake H. (2011). Hypoxia inducible factor expression in intrahepatic cholangiocarcinoma. Hepatogastroenterology.

[99] Le Calvé B., Griveau A., Vindrieux D., Maréchal R., Wiel C., Svrcek M., Gout J., Azzi L., Payen L., Cros J. (2016). Lysyl oxidase family activity promotes resistance of pancreatic ductal adenocarcinoma to chemotherapy by limiting the intratumoral anticancer drug distribution. Oncotarget.

[100] Brabletz T., Jung A., Spaderna S., Hlubek F., Kirchner T. (2005). Opinion: Migrating cancer stem cells—An integrated concept of malignant tumour progression. Nat. Rev. Cancer.

[101] Hermann P.C., Huber S.L., Herrler T., Aicher A., Ellwart J.W., Guba M., Bruns C.J., Heeschen C. (2007). Distinct populations of cancer stem cells determine tumor growth and metastatic activity in human pancreatic cancer. Cell Stem Cell.

[102] Reim F., Dombrowski Y., Ritter C., Buttmann M., Häusler S., Ossadnik M., Krockenberger M., Beier D., Beier C.P., Dietl J. (2009). Immunoselection of breast and ovarian cancer cells with trastuzumab and natural killer cells: Selective escape of CD44high/CD24low/HER2low breast cancer stem cells. Cancer Res..

[103] Al-Hajj M., Wicha M.S., Benito-Hernandez A., Morrison S.J., Clarke M.F. (2003). Prospective identification of tumorigenic breast cancer cells. Proc. Natl. Acad. Sci. USA.

[104] Li C., Heidt D.G., Dalerba P., Burant C.F., Zhang L., Adsay V., Wicha M., Clarke M.F., Simeone D.M. (2007). Identification of pancreatic cancer stem cells. Cancer Res..

[105] Oikawa T., Wauthier E., Dinh T.A., Selitsky S.R., Reyna-Neyra A., Carpino G., Levine R., Cardinale V., Klimstra D., Gaudio E. (2015). Model of fibrolamellar hepatocellular carcinomas reveals striking enrichment in cancer stem cells. Nat. Commun..

[106] Cardinale V., Renzi A., Carpino G., Torrice A., Bragazzi M.C., Giuliante F., DeRose A.M., Fraveto A., Onori P., Napoletano C. (2015). Profiles of cancer stem cell subpopulations in cholangiocarcinomas. Am. J. Pathol..

[107] Alison M.R. (2005). Liver stem cells: Implications for hepatocarcinogenesis. Stem Cell Rev..

[108] Haraguchi N., Ishii H., Mimori K., Tanaka F., Ohkuma M., Kim H.M., Akita H., Takiuchi D., Hatano H., Nagano H. (2010). CD13 is a therapeutic target in human liver cancer stem cells. J. Clin. Investig..

[109] Lee T.K., Castilho A., Cheung V.C., Tang K.H., Ma S., Ng I.O. (2011). CD24^+^ liver tumor-initiating cells drive self-renewal and tumor initiation through STAT3-mediated NANOG regulation. Cell Stem Cell.

[110] Keeratichamroen S., Leelawat K., Thongtawee T., Narong S., Aegem U., Tujinda S., Praditphol N., Tohtong R. (2011). Expression of CD24 in cholangiocarcinoma cells is associated with disease progression and reduced patient survival. Int. J. Oncol..

[111] Leelawat K., Keeratichamroen S., Leelawat S., Tohtong R. (2013). CD24 induces the invasion of cholangiocarcinoma cells by upregulating CXCR4 and increasing the phosphorylation of ERK1/2. Oncol. Lett..

[112] Takaishi S., Okumura T., Tu S., Wang S.S., Shibata W., Vigneshwaran R., Gordon S.A., Shimada Y., Wang T.C. (2009). Identification of gastric cancer stem cells using the cell surface marker CD44. Stem Cells.

[113] Gu M.J., Jang B.I. (2014). Clinicopathologic significance of Sox2, CD44 and CD44v6 expression in intrahepatic cholangiocarcinoma. Pathol. Oncol. Res..

[114] Yang Z.F., Ho D.W., Ng M.N., Lau C.K., Yu W.C., Ngai P., Chu P.W., Lam C.T., Poon R.T., Fan S.T. (2008). Significance of CD90^+^ cancer stem cells in human liver cancer. Cancer Cell.

[115] Sukowati C.H., Anfuso B., Torre G., Francalanci P., Crocè L.S., Tiribelli C. (2013). The expression of CD90/Thy-1 in hepatocellular carcinoma: An in vivo and in vitro study. PLoS ONE.

[116] Wang M., Xiao J., Jiang J., Qin R. (2011). CD133 and ALDH may be the molecular markers of cholangiocarcinoma stem cells. Int. J. Cancer.

[117] Shimada M., Sugimoto K., Iwahashi S., Utsunomiya T., Morine Y., Imura S., Ikemoto T. (2010). CD133 expression is a potential prognostic indicator in intrahepatic cholangiocarcinoma. J. Gastroenterol..

[118] Fan L., He F., Liu H., Zhu J., Liu Y., Yin Z., Wang L., Guo Y., Wang Z., Yan Q. (2011). CD133: A potential indicator for differentiation and prognosis of human cholangiocarcinoma. BMC Cancer.

[119] Wang M., Xiao J., Shen M., Yahong Y., Tian R., Zhu F., Jiang J., Du Z., Hu J., Liu W. (2011). Isolation and characterization of tumorigenic extrahepatic cholangiocarcinoma cells with stem cell-like properties. Int. J. Cancer.

[120] Govaere O., Wouters J., Petz M., Vandewynckel Y.P., Van den Eynde K., Van den Broeck A., Verhulst S., Dollé L., Gremeaux L., Ceulemans A. (2016). Laminin-332 sustains chemoresistance and quiescence as part of the human hepatic cancer stem cell niche. J. Hepatol..

[121] Cavalloni G., Peraldo-Neia C., Varamo C., Casorzo L., Dell’Aglio C., Bernabei P., Chiorino G., Aglietta M., Leone F. (2016). Establishment and characterization of a human intrahepatic cholangiocarcinoma cell line derived from an Italian patient. Tumour Biol..

[122] Bourguignon L.Y., Wong G., Earle C., Chen L. (2012). Hyaluronan-CD44v3 interaction with Oct4-Sox2-Nanog promotes miR-302 expression leading to self-renewal, clonal formation, and cisplatin resistance in cancer stem cells from head and neck squamous cell carcinoma. J. Biol. Chem..

[123] Kobayashi S., Werneburg N.W., Bronk S.F., Kaufmann S.H., Gores G.J. (2005). Interleukin-6 contributes to Mcl-1 up-regulation and TRAIL resistance via an Akt-signaling pathway in cholangiocarcinoma cells. Gastroenterology.

[124] Meng F., Yamagiwa Y., Ueno Y., Patel T. (2006). Over-expression of interleukin-6 enhances cell survival and transformed cell growth in human malignant cholangiocytes. J. Hepatol..

[125] Park J., Tadlock L., Gores G.J., Patel T. (1999). Inhibition of interleukin 6-mediated mitogen-activated protein kinase activation attenuates growth of a cholangiocarcinoma cell line. Hepatology.

[126] Trouillas M., Saucourt C., Guillotin B., Gauthereau X., Taupin J.L., Moreau J.F., Boeuf H. (2009). The LIF cytokine: Towards adulthood. Eur. Cytokine Netw..

[127] Mathieu M.E., Saucourt C., Mournetas V., Gauthereau X., Thézé N., Praloran V., Thiébaud P., Bœuf H. (2012). LIF-dependent signaling: New pieces in the Lego. Stem Cell Rev..

[128] Aghajanova L. (2004). Leukemia inhibitory factor and human embryo implantation. Ann. N. Y. Acad. Sci..

[129] Mahic M., Kalland M.E., Aandahl E.M., Torgersen K.M., Taskén K. (2008). Human naturally occurring and adaptive regulatory T cells secrete high levels of leukaemia inhibitory factor upon activation. Scand. J. Immunol..

[130] Sims N.A., Johnson R.W. (2012). Leukemia inhibitory factor: A paracrine mediator of bone metabolism. Growth Factors.

[131] Oskowitz A.Z., Lu J., Penfornis P., Ylostalo J., McBride J., Flemington E.K., Prockop D.J., Pochampally R. (2008). Human multipotent stromal cells from bone marrow and microRNA: Regulation of differentiation and leukemia inhibitory factor expression. Proc. Natl. Acad. Sci. USA.

[132] Heinrich P.C., Behrmann I., Müller-Newen G., Schaper F., Graeve L. (1998). Interleukin-6-type cytokine signalling through the gp130/Jak/STAT pathway. Biochem. J..

[133] Giese B., Roderburg C., Sommerauer M., Wortmann S.B., Metz S., Heinrich P.C., Müller-Newen G. (2005). Dimerization of the cytokine receptors gp130 and LIFR analysed in single cells. J. Cell Sci..

[134] Zouein F.A., Kurdi M., Booz G.W. (2013). LIF and the heart: Just another brick in the wall?. Eur. Cytokine Netw..

[135] Heinrich P.C., Behrmann I., Haan S., Hermanns H.M., Müller-Newen G., Schaper F. (2003). Principles of interleukin (IL)-6-type cytokine signalling and its regulation. Biochem. J..

[136] Morton S.D., Cadamuro M., Brivio S., Vismara M., Stecca T., Massani M., Bassi N., Furlanetto A., Joplin R.E., Floreani A. (2015). Leukemia inhibitory factor protects cholangiocarcinoma cells from drug-induced apoptosis via a PI3K/AKT-dependent Mcl-1 activation. Oncotarget.

[137] Kellokumpu-Lehtinen P., Talpaz M., Harris D., Van Q., Kurzrock R., Estrov Z. (1996). Leukemia-inhibitory factor stimulates breast, kidney and prostate cancer cell proliferation by paracrine and autocrine pathways. Int. J. Cancer.

[138] Kamohara H., Sakamoto K., Ishiko T., Masuda Y., Abe T., Ogawa M. (1997). Leukemia inhibitory factor induces apoptosis and proliferation of human carcinoma cells through different oncogene pathways. Int. J. Cancer.

[139] Andrae J., Gallini R., Betsholtz C. (2008). Role of platelet-derived growth factors in physiology and medicine. Genes Dev..

[140] Heldin C.H., Westermark B. (1999). Mechanism of action and in vivo role of platelet-derived growth factor. Physiol. Rev..

[141] Jechlinger M., Sommer A., Moriggl R., Seither P., Kraut N., Capodiecci P., Donovan M., Cordon-Cardo C., Beug H., Grünert S. (2006). Autocrine PDGFR signaling promotes mammary cancer metastasis. J. Clin. Investig..

[142] Wang Z., Ahmad A., Li Y., Kong D., Azmi A.S., Banerjee S., Sarkar F.H. (2010). Emerging roles of PDGF-D signaling pathway in tumor development and progression. Biochim. Biophys. Acta.

[143] Fingas C.D., Bronk S.F., Werneburg N.W., Mott J.L., Guicciardi M.E., Cazanave S.C., Mertens J.C., Sirica A.E., Gores G.J. (2011). Myofibroblast-derived PDGF-BB promotes Hedgehog survival signaling in cholangiocarcinoma cells. Hepatology.

[144] Pietras K., Sjöblom T., Rubin K., Heldin C.H., Ostman A. (2003). PDGF receptors as cancer drug targets. Cancer Cell.

[145] Strazzabosco M., Fabris L. (2012). Development of the bile ducts: Essentials for the clinical hepatologist. J. Hepatol..

[146] Clevers H. (2006). Wnt/β-catenin signaling in development and disease. Cell.

[147] Spirli C., Locatelli L., Morell C.M., Fiorotto R., Morton S.D., Cadamuro M., Fabris L., Strazzabosco M. (2013). Protein kinase A-dependent pSer^675^-β-catenin, a novel signaling defect in a mouse model of congenital hepatic fibrosis. Hepatology.

[148] Shen D.Y., Zhang W., Zeng X., Liu C.Q. (2013). Inhibition of Wnt/β-catenin signaling downregulates P-glycoprotein and reverses multi-drug resistance of cholangiocarcinoma. Cancer Sci..

[149] Huang G.L., Shen D.Y., Cai C.F., Zhang Q.Y., Ren H.Y., Chen Q.X. (2015). β-escin reverses multidrug resistance through inhibition of the GSK3β/β-catenin pathway in cholangiocarcinoma. World J. Gastroenterol..

[150] Wang W., Zhong W., Yuan J., Yan C., Hu S., Tong Y., Mao Y., Hu T., Zhang B., Song G. (2015). Involvement of Wnt/β-catenin signaling in the mesenchymal stem cells promote metastatic growth and chemoresistance of cholangiocarcinoma. Oncotarget.

[151] Okabe H., Yang J., Sylakowski K., Yovchev M., Miyagawa Y., Nagarajan S., Chikina M., Thompson M., Oertel M., Baba H. (2016). Wnt signaling regulates hepatobiliary repair following cholestatic liver injury in mice. Hepatology.

[152] Lamar J.M., Stern P., Liu H., Schindler J.W., Jiang Z.G., Hynes R.O. (2012). The Hippo pathway target, YAP, promotes metastasis through its TEAD-interaction domain. Proc. Natl. Acad. Sci. USA.

[153] Piccolo S., Cordenonsi M., Dupont S. (2013). Molecular pathways: YAP and TAZ take center stage in organ growth and tumorigenesis. Clin. Cancer Res..

[154] Piccolo S., Dupont S., Cordenonsi M. (2014). The biology of YAP/TAZ: Hippo signaling and beyond. Physiol. Rev..

[155] Azzolin L., Panciera T., Soligo S., Enzo E., Bicciato S., Dupont S., Bresolin S., Frasson C., Basso G., Guzzardo V. (2014). YAP/TAZ incorporation in the β-catenin destruction complex orchestrates the Wnt response. Cell.

[156] Wang K.C., Yeh Y.T., Nguyen P., Limqueco E., Lopez J., Thorossian S., Guan K.L., Li Y.J., Chien S. (2016). Flow-dependent YAP/TAZ activities regulate endothelial phenotypes and atherosclerosis. Proc. Natl. Acad. Sci. USA.

[157] Zanconato F., Cordenonsi M., Piccolo S. (2016). YAP/TAZ at the Roots of Cancer. Cancer Cell.

[158] Marti P., Stein C., Blumer T., Abraham Y., Dill M.T., Pikiolek M., Orsini V., Jurisic G., Megel P., Makowska Z. (2015). YAP promotes proliferation, chemoresistance, and angiogenesis in human cholangiocarcinoma through TEAD transcription factors. Hepatology.

[159] Morell C.M., Fiorotto R., Fabris L., Strazzabosco M. (2013). Notch signalling beyond liver development: Emerging concepts in liver repair and oncogenesis. Clin. Res. Hepatol. Gastroenterol..

[160] Espinoza I., Pochampally R., Xing F., Watabe K., Miele L. (2013). Notch signaling: Targeting cancer stem cells and epithelial-to-mesenchymal transition. OncoTargets Ther..

[161] Geisler F., Strazzabosco M. (2015). Emerging roles of Notch signaling in liver disease. Hepatology.

[162] Villanueva A., Alsinet C., Yanger K., Hoshida Y., Zong Y., Toffanin S., Rodriguez-Carunchio L., Solé M., Thung S., Stanger B.Z. (2012). Notch signaling is activated in human hepatocellular carcinoma and induces tumor formation in mice. Gastroenterology.

[163] Strazzabosco M., Fabris L. (2012). Notch signaling in hepatocellular carcinoma: Guilty in association!. Gastroenterology.

[164] Fan B., Malato Y., Calvisi D.F., Naqvi S., Razumilava N., Ribback S., Gores G.J., Dombrowski F., Evert M., Chen X. (2012). Cholangiocarcinomas can originate from hepatocytes in mice. J. Clin. Investig..

[165] Zender S., Nickeleit I., Wuestefeld T., Sörensen I., Dauch D., Bozko P., El-Khatib M., Geffers R., Bektas H., Manns M.P. (2013). A critical role for notch signaling in the formation of cholangiocellular carcinomas. Cancer Cell.

[166] Wu W.R., Zhang R., Shi X.D., Zhu M.S., Xu L.B., Zeng H., Liu C. (2014). Notch1 is overexpressed in human intrahepatic cholangiocarcinoma and is associated with its proliferation, invasiveness and sensitivity to 5-fluorouracil in vitro. Oncol. Rep..

[167] Omenetti A., Diehl A.M. (2011). Hedgehog signaling in cholangiocytes. Curr. Opin. Gastroenterol..

[168] Riedlinger D., Bahra M., Boas-Knoop S., Lippert S., Bradtmöller M., Guse K., Seehofer D., Bova R., Sauer I.M., Neuhaus P. (2014). Hedgehog pathway as a potential treatment target in human cholangiocarcinoma. J. Hepatobiliary Pancreat. Sci..

[169] Kurita S., Mott J.L., Almada L.L., Bronk S.F., Werneburg N.W., Sun S.Y., Roberts L.R., Fernandez-Zapico M.E., Gores G.J. (2010). GLI3-dependent repression of DR4 mediates hedgehog antagonism of TRAIL-induced apoptosis. Oncogene.

[170] Razumilava N., Bronk S.F., Smoot R.L., Fingas C.D., Werneburg N.W., Roberts L.R., Mott J.L. (2012). miR-25 targets TNF-related apoptosis inducing ligand (TRAIL) death receptor-4 and promotes apoptosis resistance in cholangiocarcinoma. Hepatology.

[171] Fingas C.D., Mertens J.C., Razumilava N., Sydor S., Bronk S.F., Christensen J.D., Rizvi S.H., Canbay A., Treckmann J.W., Paul A. (2013). Polo-like kinase 2 is a mediator of hedgehog survival signaling in cholangiocarcinoma. Hepatology.

[172] El Khatib M., Kalnytska A., Palagani V., Kossatz U., Manns M.P., Malek N.P., Wilkens L., Plentz R.R. (2013). Inhibition of hedgehog signaling attenuates carcinogenesis in vitro and increases necrosis of cholangiocellular carcinoma. Hepatology.

[173] Hong I.S., Lee H.Y., Nam J.S. (2015). Cancer stem cells: The “Achille’s heel” of chemo-resistant tumors. Recent Pat. Anticancer Drug Discov..

[174] Swaminathan S.K., Roger E., Toti U., Niu L., Ohlfest J.R., Panyam J. (2013). CD133-targeted paclitaxel delivery inhibits local tumor recurrence in a mouse model of breast cancer. J. Control. Release.

[175] Marangoni E., Lecomte N., Durand L., de Pinieux G., Decaudin D., Chomienne C., Smadja-Joffe F., Poupon M.F. (2009). CD44 targeting reduces tumour growth and prevents post-chemotherapy relapse of human breast cancers xenografts. Br. J. Cancer.

[176] Edris B., Weiskopf K., Volkmer A.K., Volkmer J.P., Willingham S.B., Contreras-Trujillo H., Liu J., Majeti R., West R.B., Fletcher J.A. (2012). Antibody therapy targeting the CD47 protein is effective in a model of aggressive metastatic leiomyosarcoma. Proc. Natl. Acad. Sci. USA.

